# Intravenous Vitamin K1 for the Correction of Prolonged Prothrombin Times in Non-Bleeding Critically Ill Patients: A Prospective Observational Study

**DOI:** 10.3390/nu13082580

**Published:** 2021-07-27

**Authors:** Sofia Dahlberg, Ulf Schött, Emilia Ängeby Eriksson, Yllnor Tahirsylaj, Leon Schurgers, Thomas Kander

**Affiliations:** 1 Thoracic Sugery, Department of Clinical Sciences, Lund University, Skane University Hospital, 22184 Lund, Sweden; 2 Anaesthesia & Intensive Care, Department of Clinical Sciences, Lund University, Skane University Hospital, 22184 Lund, Sweden; ulf.schott@med.lu.se (U.S.); thomas.kander@med.lu.se (T.K.); 3 Department of Clinical Sciences, Lund University, 22184 Lund, Sweden; emiliaeriksson1995@hotmail.com (E.Ä.E.); lak15yta@student.lu.se (Y.T.); 4 Department of Biochemistry, Cardiovascular Research Institute, 6200 MD Maastricht, The Netherlands; l.schurgers@maastrichtuniversity.nl

**Keywords:** prothrombin, vitamin K, Gla protein, PIVKA-II, dp-ucMGP, intensive care

## Abstract

The aim of this study was to evaluate the effects of vitamin K1 on various vitamin K-dependent proteins in critically ill patients with prolonged Owren PT. We included critically ill non-bleeding adult patients without liver failure or anticoagulation treatment, with Owren PT > 1.2, who were prescribed intravenous vitamin K1. Blood was drawn at baseline and at 20–28 h after vitamin K1 administration. At both time points, we measured various vitamin K-dependent proteins and coagulation assays. ClinicalTrials.gov; Identifier: NTC3782025. In total, 52 patients were included. Intravenous vitamin K1 reduced Owren PT, Quick PT, protein induced by vitamin K absence/antagonist-II and desphospho-uncarboxylated matrix Gla protein (dp-ucMGP), but not to normal levels. Concomitantly, there were increases in thrombin generation and the activity of coagulation factors II, VII, IX and X that was only counteracted with a small increase in Protein C activity. In conclusion, the results suggest that vitamin K1 strengthens coagulation as measured by PT decrease and increases in the activity of vitamin K-dependent clotting factors and thrombin generation. The decreased dp-ucMGP, and its potential positive short- and long-term non-coagulative effects, merits further research.

## 1. Introduction

Coagulation abnormalities frequently occur in critically ill patients and affect mortality [1,2]. Opinions differ on how laboratory signs of increased bleeding diathesis in non-bleeding patients should be treated. There are studies suggesting that there is a benefit in treating even mildly prolonged prothrombin times (PTs) in non-bleeding critically ill patients or prior to invasive procedures [3,4,5], but high-quality data is lacking and there is no consensus on the issue of treating a prolonged PT, possibly induced by a vitamin K deficiency, with intravenous vitamin K1 [2]. The term vitamin K identifies a series of compounds deriving from 2-methyl-1,4-naphthoquinone. Vitamin K is fundamental for coagulation as it takes part in the activation processes of some coagulation factors such as factors II, VII, IX, X, and protein C and protein S [6].

Vitamin K deficiency in hospitalized patients can be misdiagnosed as disseminated intravascular coagulopathy and vitamin K1 has been proposed to reverse prolonged PTs in non-emergent situations [7]. To the best of our knowledge, there have not been any thorough investigations with regard to vitamin K1 administration in non-bleeding adult critically ill patients and its effects on vitamin K-dependent proteins (VKDPs) (Factor [F] II, FVII, FIX, FX, protein C and protein S), thrombin generation assay (TGA) and rotational thromboelastometry (ROTEM). In addition to strengthening the coagulation system, vitamin K has been suggested to have beneficial pleiotropic effects that extend beyond activation of coagulation factors [8]. These effects are thought to be mediated through several extrahepatic vitamin K-dependent proteins, with potential involvement in protection against cardiovascular [9], neurodegenerative [10] and metabolic diseases as well as cancer [8,11].

The discovery of vitamin K’s role in post-translational gamma-carboxylation of proteins in the 1970s resulted in the identification of the vitamin K cycle, which is a series of enzymatic modifications vitamin K undergoes in order to support carboxylation [12]. The vitamin K cycle has been detected in most tissues in the body, able to carboxylate several so-called extrahepatic VKDPs. Osteocalcin was the first extrahepatic VKDP to be detected in the 1980s [13]. Since then, several others have been described, such as Matrix Gla protein (MGP), Growth Arrest Specific 6 protein (Gas6) and Gla Rich Protein (GRP).

The activity of some of the VKDPs can be estimated by measuring the levels of their respective uncarboxylated proteins, where high levels correspond to low activity [14,15,16,17]. Uncarboxylated fractions of VKDPs cannot bind calcium and are therefore unable to interact with phospholipid surfaces and specific receptors. High levels of uncarboxylated prothrombin (also known as protein induced by vitamin K absence/antagonist-II [PIVKA-II]) have been observed in critically ill patients at both the time of admission and several days after intensive care unit (ICU) admission [16,17,18], which indicates that critically ill patients might benefit from vitamin K1 administration.

The aim of this study was to evaluate the effect of vitamin K1 on a wide range of coagulation assays in non-bleeding critically ill patients with spontaneously prolonged Owren PT. In addition, the activity of PIVKA-II and desphospho-uncarboxylated matrix Gla protein (dp-ucMGP) were analysed before and after vitamin K1 administration.

## 2. Materials and Methods

### 2.1. Study Design

This prospective observational study was performed at Lund University Hospital, Sweden, and was approved by the Lund Regional Ethical Review Board (DNR 2018/1010) and registered on ClinicalTrials.gov (Identifier: NTC3782025). Written and informed consent was obtained from all participating patients either prior to the sampling or, in cases where this was not possible, afterwards at the earliest moment when the patient was competent to make decisions. Non-bleeding patients aged 18 years or older who were admitted to the ICU or the postoperative care unit after all-day surgery (e.g., pancreatico-duodenectomy or oesophageal resection) with Owren PT > 1.2 and who were prescribed 10 mg intravenous vitamin K1 (phytomenadione Konakion Novum^®^, Cheplapharm Arzneimittel GmbH, Greifswald, Germany) with a slow injection during office hours were eligible. The dose of 10 mg was decided by the responsible physician.

The exclusion criteria comprised absence of written and informed consent, hepatocellular carcinoma, liver resection in the past 6 months, treatment with warfarin or novel oral anticoagulants (NOAC), known pre-existing coagulopathy and administration of vitamin K1 in the past 36 h. Patients were recruited during two separate trial periods: February to April 2019 and September 2019 to March 2020. All the included patients were treated according to the standard of care at the ICU and the postoperative care unit.

### 2.2. Blood Sampling and Laboratory Analysis

Arterial blood samples were taken within 6 h prior to vitamin K1 administration and then again at 20 to 28 h after vitamin K1 administration. All blood samples were collected using the BD vacutainer system (BD, Plymouth, UK). Owren PT, Quick PT, FII, FVII, FIX, FX, protein C and protein S activity, dp-ucMGP and ROTEM (ex-TEM assay) were analysed in citrated tubes. A sample with carbohydrate-deficient transferrin was obtained for TGA, and a serum tube sample was used to assess PIVKA-II.

All blood samples except one citrate tube used for ROTEM (ex-TEM assay) and one used for Owren PT were centrifuged immediately according to the instructions for each analysis. The plasma and serum were then stored at −85 °C until further analysis.

### 2.3. Standard Coagulation Assays

Owren PT is sensitive to changes in FII, FVII and FX and was measured at the central accredited laboratory at the hospital using a combined thromboplastin reagent (Owren PT, Medirox, Sweden) and calibrated using reference samples with certified international normalized ratios (INRs) from Equalis (Uppsala, Sweden). The reference range for the Owren PT was 0.9–1.2, with a coefficient of variation (CV) < 4%.

Quick PT is sensitive to changes in fibrinogen, FII, FV, FVII and FX and the results were expressed in seconds. The reagent is a mixture of tissue factor, thromboplastin and calcium. The reference interval was 10–13 s, with a CV < 4%.

APTT is sensitive to changes in FII, FV, FVIII–XII and fibrinogen and was measured at the central accredited laboratory at the hospital using a reagent (Actin FSL, Siemens Healthcare Diagnostics, Marburg, Germany) that activates the intrinsic pathway and phospholipids. The results were expressed in seconds, and the reference interval was 26–33 s, with a CV < 4%.

Fibrinogen was measured with a photometric assay (Dade Thrombin, Siemens). Thrombin (50 U/mL) was added in excess to plasma samples. Clotting time was recorded with an automated coagulometer (Sysmex CS 5100, Siemens, Marburg, Germany) and compared to clotting times with known fibrinogen concentrations. According to the manufacturer, the reference interval for fibrinogen was 2–4 g/L with a CV of 5%.

### 2.4. Vitamin K-Dependent Coagulation Factor Activities

Coagulation factor activity was determined with the Thromborel S reagent (Siemens), and dilutions were made in factor-deficient plasma in the BCS-XP Coagulation analyser (Siemens) at our accredited Coagulation Laboratory, Malmö. The results were reported in kIU/L and the reference intervals were as follows: FII, 0.70–1.50 kIU/L; FVII, 0.40–1.60 kIU/L; FIX, 0.80–1.50 kIU/L; FX, 0.70–1.54 kIU/L; protein C, 0.70–1.30 kIU/L; and protein S, 0.65–1.40 kIU/L. All analytes had CVs < 8%.

### 2.5. Thrombin Generation Assay

The TGA was performed using Ceveron alpha^®^ (Technoclone, Vienna, Austria), an automatic system which measures the formation of thrombin over time with a fluorescent substrate. Coagulation was activated in vitro by tissue factor (TF) in the presence of phospholipids. The TGA then provided a curve of thrombin generation over time. The results included a lag time (the duration before the thrombin burst) in seconds (s), endogenous thrombin potential (the area under the curve) in nM × min, and peak thrombin (peak concentration of thrombin formed) in nM. The test was done with two different reagents: TGA RB, which has a low concentration of phospholipids and TF, and TGA RC High, which has a high concentration of TF. The reference intervals with the TGA RC High reagent were: lag time, 2.2–3.4 s; peak height, 67–459 nM; and area under curve (AUC), 1195–2568 nM × min. The reference intervals for TGA RB were: lag time, 5.2–11.3 s; peak height, 36–372 nM; and AUC, 1538–2652 nM × min. CVs for the respective parameters have been published previously [19].

### 2.6. ROTEM

ROTEM (EXTEM assay) (Pentapharm GmbH, Munich, Germany) was used to measure clot elasticity and clot formation. The analyses were performed according to the manufacturer’s instructions. The reference values were 38–79 s for clotting time (CT), 34–159 s for clot formation time (CFT), 63–83° for the α-angle, 50–77 mm for maximum clot firmness (MCF) and 0–18% for maximum lysis (ML). All samples for ROTEM were analysed in duplicate, and mean values were calculated. CVs for the respective parameters have been published previously [19].

### 2.7. dp-ucMGP and PIVKA-II

The analyses of dp-ucMGP and PIVKA-II were performed at the Coagulation Profile Laboratory (Maastricht, The Netherlands). Plasma dp-ucMGP levels were determined using the commercially available IVD CE-marked chemiluminescent InaKtif MGP assay on the IDS-iSYS system (IDS, Boldon, UK). In brief, samples were exposed to magnetic particles coated with monoclonal antibodies against dp-MGP and uc-MGP. Trigger reagents were then added, resulting in light emission that was directly proportional to the level of dp-ucMGP in the sample. The within-run and total variations of this assay were 0.8–6.2% and 3.0–8.2%, respectively. The assay quantitation range was between 200 and 12,000 pmol/L and was linear up to 11,651 pmol/L. The dp-ucMGP assay has been validated previously by various researchers [20]. As for the upper reference value, this is somewhat arbitrary. Biochemically, values of inactive vitamin K-dependent proteins should be below detection (i.e., zero). However, subclinical vitamin K deficiency is common in the population. Therefore, the upper reference value was set to 300 pmol/L [21]. 

Circulating serum PIVKA-II levels were measured using a conformation-specific monoclonal antibody in an enzyme-linked immunosorbent assay-based non-commercial serum test. Results were expressed as arbitrary units per milliliter (AU/mL). Using electrophoretic techniques, 1 AU was determined to be equivalent to 1 mg of purified uncarboxylated FII. The detection limit was 0.15 AU/mL, designed only to detect very high PIVKA-II levels.

### 2.8. Clinical Data

Clinical data were obtained from the patient data management system (ICCA, Philips, Amsterdam, The Netherlands) or from the electronic charts. The Sequential Organ Failure Assessment (SOFA) score is a well-established daily score for the number of failing organ systems in critically ill patients [22]. Simplified Acute Physiology Score 3 (SAPS 3), an updated severity score for critically ill patients [23], was obtained from the local quality register (PASIVA, Otimo Data AB, Kalmar, Sweden).

### 2.9. Statistical Analysis

Sample size calculation was performed using G*Power version 3.1 (Heinrich Heine Universität, Düsseldorf, Germany) for the Wilcoxon signed-rank test and was based on a pilot study from our department where 10 mg Konakion Novum^®^ was given intravenously to non-bleeding critically ill patients with prolonged Owren PT. The effect size in that study was 0.48 and was based on changes in Owren PT. With a two-tailed α-value of 0.05 and 90% power, the target sample size was set to 50 patients for this study.

Data were processed using IBM SPSS for Windows, version 27.0 (SPSS Inc., Chicago, IL, USA).

Normality tests were used to test for normal distribution and continuous variables were summarized accordingly, and numbers were given along with percentages. The differences in the samples before and after vitamin K1 administration were analysed using the two-tailed Wilcoxon signed-rank test, and the comparison of changes in the sensitivity analyses was performed using the Mann–Whitney test. The significance level was set to 0.05 for all statistical tests.

## 3. Results

### 3.1. Baseline

**Figure 1:**
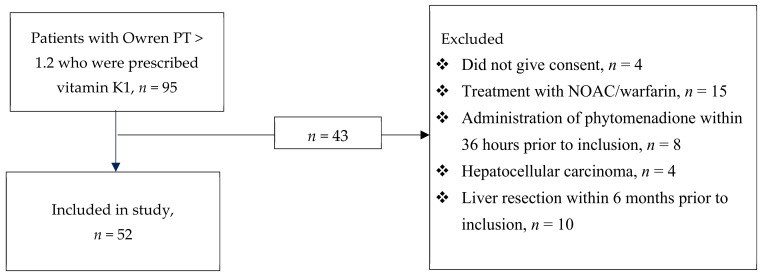
Consort diagram. PT: prothrombin time, NOAC: novel oral anticoagulant.

In total, 95 patients were eligible for inclusion. None of these patients had any anaphylactoid/anaphylactic reaction. After the exclusion of 43 patients that met at least one of the exclusion criteria, 52 patients remained and were included in the study (Figure 1). Patient characteristics are shown in Table 1. The sample was predominately male (69%), and the median age was 68 years (range: 55–74 years). The most common ICU diagnosis was sepsis, followed by postoperative care and cardiovascular disease. The median SOFA score at inclusion was 7 (range: 5–11), and the median SAPS 3 at admission was 65 (range: 52–74). No adverse reactions to vitamin K1 were reported. A post-hoc power analysis was performed, and the observed power was 91%.

Detailed results of the assays performed before and after vitamin K1 administration are presented in Table 2.

### 3.2. Standard Coagulation Assays

Detailed results in Table 2. The median Owren PT before vitamin K1 administration was 1.4 (range: 1.3–1.6) and decreased to 1.3 (range: 1.2–1.4, *p* < 0.001) after vitamin K1 administration. A similar decrease was noted in Quick PT, whereas APTT remained unchanged. Fibrinogen concentrations increased (*p* < 0.001).

### 3.3. Vitamin K-Dependent Coagulation Factor Activity

Detailed results in Table 2. The activities of FII, FVII, FIX and FX all significantly increased (*p* < 0.001). It should be noted that FII and FX were below the normal reference range before vitamin K1 administration, whereas fibrinogen plasma levels were above the reference range. All coagulation factors were within their normal ranges after vitamin K1 administration.

The activities of protein C and protein S were both slightly below the normal reference range before vitamin K1 administration. Protein C activity remained unchanged (*p* = 0.11) after vitamin K1 administration, whereas protein S activity increased (*p* = 0.024), but was still just below its normal reference range.

### 3.4. Thrombin Generation Assay

Thrombin generation as measured by TGA RB and TGA RC increased (*p* = 0.006 and *p* = 0.005, respectively), and the median values were always within the normal reference ranges.

### 3.5. ROTEM

Except for CT, which was slightly prolonged before vitamin K1 administration, all ROTEM parameters had median values within the normal reference ranges at all times. MCF increased after vitamin K1 administration (*p* = 0.016), and the remaining ROTEM parameters were unchanged.

### 3.6. dp-ucMGP and PIVKA-II 

The median level of dp-ucMGP decreased after vitamin K1 administration (*p* < 0.001). The median dp-ucMGP was above the normal reference interval both before and after vitamin K1 administration. Detailed results in Table 2. PIVKA-II was below the detection limit in nearly all patients (44/50, 88%) both before and after vitamin K1 administration. In the patients with detectable PIVKA-II levels (*n* = 6), a decrease in response to vitamin K1 was seen (*p* = 0.031).

### 3.7. Additional Analyses

When comparing laboratory values and SOFA score before and after vitamin K1 administration, haemoglobin and SOFA score decreased (*p* = 0.002 and *p* = 0.005, respectively), whereas C-reactive protein (CRP) increased (*p* = 0.01). Leukocytes and platelets were unchanged (*p* = 0.73 and *p* = 0.23, respectively), as shown in Table 2.

### 3.8. Sensitivity Analysis

To investigate whether the changes in coagulation parameters and vitamin K-dependent protein activity between the two time points could be explained by a general improvement in patient condition over time, patients were divided into two groups based on whether their SOFA score decreased (*n* = 27) or increased (or remained unchanged, *n* = 25) between the two time points. CRP was not used for the separation in two groups as the response time for CRP is prolonged and for SOFA score is instant [24].

The detailed results of this analysis are shown in Table 3. In short, patients in the group with the decreasing SOFA score (indicating clinical improvement) did not demonstrate better restoration of the measured proteins as compared to the group with increased or unchanged SOFA score. The delta changes between the time points also demonstrated a stronger return to a normal Owren PT in the group with increased or unchanged SOFA score.

## 4. Discussion

In this prospective single-centre observational study, which included non-bleeding critically ill patients with increased Owren PT, their coagulation status as measured by several assays improved overall in response to vitamin K1. Simultaneously, dp-ucMGP and PIVKA-II decreased after vitamin K1 administration but not to normal levels, suggesting only a partial restoration of vitamin K status. Protein C and protein S, which have anticoagulative properties, did not increase as convincingly as the pro-coagulative VKDPs, indicating that vitamin K1 administration elicited a predominantly pro-coagulative response. In a post-hoc sensitivity analysis, patients demonstrating clinical improvement did not show a stronger reversal in the levels of the measured proteins, indicating that the strengthened coagulation after vitamin K1 administration was not caused by reduced coagulopathy over time. 

Although vitamin K1 is routinely given to critically ill patients with increased PT, the effect of this treatment has, to the best of our knowledge, never been thoroughly examined in prospective cohort studies in non-warfarin anticoagulated adults [2]. In a retrospective study from our department, vitamin K1 produced a slightly larger decrease in PT as compared to non-supplemented patients in a control group [7]. Furthermore, in the present study, we demonstrated that vitamin K1 administration corrected coagulation in patients with increased Owren PT (i.e., in the absence of warfarin, NOAC or liver failure) and may also affect the production and carboxylation of VKDPs, thus exhibiting the potential for other positive effects.

A partial cause for the prolonged PT at baseline in the present study may have been vitamin K deficiency, indicated by the relative low vitamin K1 nutritional administration between the sampling occasions as compared to the United States Food and Drug Administration recommendations of daily intake (Table 2). Even though the nutritional support between the sample occasions were not always in a stable steady state, the relative deficiency is an indication for suboptimal vitamin K administration during critical illness. The pathophysiology underlying vitamin K deficiency in hospitalised patients is multifactorial, and contributing factors include insufficient supply (malnutrition, prolonged intravenous nutrition), malabsorption (bowel inflammation, gastric retention, sepsis or ischemic induced intestinal wall damage), drugs and increased vitamin K usage seen during acute illness [25]. It is probable that insufficient supply and malabsorption are the main culprits for vitamin K deficiency in critically ill patients.

Although none of the patients in the present study had any anaphylactoid/anaphylactic reactions, phytomenadione has a black box warning due to severe anaphylaxis when given IV that may cause shock, respiratory arrest, and cardiac arrest [26]. Therefore, a slow injection or infusion is recommended and signs for anaphylaxis highlighted, treated and injection/ infusion stopped. National guidelines differ between different countries for how to administer phytomenadione and should be followed. 

PIVKA-II levels were below the detection limit for most patients, depending on a noncommercial test adapted to detect only high PIVKA-II levels. This contrasts with previous studies, where elevated PIVKA-II levels have been demonstrated in 82–86% of ICU and perioperative patients [14,15,16,17]. In those studies, we used a commercial method for PIVKA-II (Stago) with a much lower LOD (Limit of detection). Although elevated levels of PIVKA-II may derive from different conditions such as liver disease and vitamin K cycle defects, it may also indicate hepatic vitamin K deficiency. Elevated levels of dp-ucMGP better reflect extrahepatic vitamin K status. 

We demonstrated a significant decrease in dp-ucMGP after a high vitamin K1 intravenous administration. Wyskida et al. did not find any correlation between vitamin K1 intake through food and ucMGP in haemodialysis patients and concluded that ucMGP was a poor marker of functional vitamin K1 deficiency [27]. We used the dp-ucMGP marker in the present study and found a decrease after a high intravenous dose of vitamin K1, which is interesting as such an effect on decarboxylated MGP usually is referred to as vitamin K2. This effect of vitamin K in higher dosages corroborates results from 2 recent studies [28,29]. Our dp-ucMGP levels are high, but in a previous study on postoperative changes after major surgery, even higher dp-ucMGP levels were measured on day 5 compared to day 1 after surgery [14]. 

MGP is an extrahepatic vitamin K-dependent protein that could have beneficial effects in critically ill patients as it acts as a calcification inhibitor [16]. To date, research has mainly focused on its potential involvement in cardiovascular disease and in uremic vascular calcification [30] and little is known about how its uncarboxylated form (dp-ucMGP) is affected by critical illness and whether it is a potential biomarker for disease or if it is involved in disease progression [31]. dp-ucMGP has been suggested as a biomarker for cardiovascular disease, and in diabetic chronic kidney disease patients, an increased dp-ucMGP level has been linked to increased mortality [30]. Cardiovascular disease is typically progressive over several years and might therefore not be regarded as relevant for the ICU population, where the duration of treatment usually ranges from days to weeks. However, pre-clinical studies have shown upregulated gene expression of MGP 24 h after myocardial infarction and peaking after 4 weeks, which might indicate that MGP is involved in or is a biomarker for post-infarction myocardial remodelling [32]. MGP has also been implicated in pulmonary disease. In two recent studies of coronavirus disease 2019 (COVID-19) patients, pronounced elevations of dp-ucMGP were demonstrated and correlated to a poorer prognosis [25,33]. Pulmonary damage and development of fibrosis may occur after long-standing mechanical ventilation and acute respiratory distress syndrome. Future studies should investigate whether vitamin K supplementation might attenuate this process. 

It should also be acknowledged that vitamin K1 is an attractive alternative to plasma transfusion without significant side effects when minor coagulation enhancement is desired and where small improvements in PT-INR may be beneficial, such as in patients with traumatic brain injuries [34] and trauma-associated coagulopathies [35] or prior to non-emergent invasive procedures [36]. Finally, the results of the present study demonstrate that it is possible to influence the activity of vitamin K-dependent proteins in critically ill patients with intravenous vitamin K1, which may have further positive long-term effects, such as in cases of sepsis, myocardial remodelling and lung fibrosis, as discussed above.

### Limitations

There are several limitations to this study that warrant mention. This is a before–after study and may have been afflicted with a time-dependent bias. Although we have tried to address this in a sensitivity analysis, a time-dependent bias cannot be completely ruled out. Furthermore, this was a single-centre study, which limits the generalisability of the results. The study population was also heterogenous with respect to diagnoses, which means that the coagulation abnormalities may have been the result of different mechanisms (such as sepsis-associated coagulopathy, dilution, or malnutrition), and some patient groups may have benefited more than others. However, as the subgroups were too small to allow for any meaningful statistical analysis, this could not be investigated. The heterogeneity of the study population may also be the reason why the effect on Owren PT was smaller than anticipated in the pilot study, used for the sample size calculation. Furthermore, no follow-up blood samples were analysed; therefore, it is unclear if the observed effects were persistent over time. Lastly, this study did not aim to study long-term effects of vitamin K1 supplementation and further studies are needed to investigate this. 

## 5. Conclusions

Although this is an explorative hypothesis-generating study in an area where there is a great paucity of knowledge, it is, to the best of our knowledge, the first study to evaluate the effect of intravenous vitamin K1 on routine coagulation status, coagulation factor activity, TGA and ROTEM analyses in critically ill patients with increased PT. Our results suggest that vitamin K1 strengthens coagulation, as evidenced by a decrease in PT and concomitant increases in vitamin K-dependent clotting factors and thrombin generation. However, there were no convincing effects on ROTEM parameters, protein C and protein S. A notable finding in this study was the effect on dp-ucMGP, whose circulating levels were decreased by one third following intravenous vitamin K1 administration, suggesting improved vitamin K status. It is still unclear whether this restoration in coagulation is accompanied by other positive effects exerted by extrahepatic vitamin K-dependent proteins, and this merits further research. 

## Figures and Tables

**Table 1:** Patient characteristics at admission.

Baseline Characteristics ^1^	Median (Interquartile Range) or No (%)
Age, years	68 (55–74)
Sex, male	36 (69)
SAPS 3 ^2^	65 (52–74)
SOFA ^3^	7 (5–11)
ICU Diagnosis	
Cancer	3 (6)
Cardiovascular	7 (13)
Respiratory	5 (10)
Septic shock	15 (29)
Surgical complication	4 (8)
Trauma	5 (10)
Postoperative care	9 (17)
Other	4 (8)
Laboratory values	
ALAT ^4^ (µkat/L)	0.65 (0.42–1.3)
ALP ^5^ (µkat/L)	0.92 (0.74–1.3)
ASAT ^6^ (µkat/L)	0.98 (0.59–1.53)
Bilirubin (µmol/L)	11.5 (9–19.5)
Creatinine (µmol/L)	79 (67–121)

^1^ Values are presented as median (IQR) for continuous variables and *n* (%) for categorical variables. ^2^ simplified Acute Physiology Score III; ^3^ sequential organ failure assessment; ^4^ alanine transaminase; ^5^ alkaline phosphatase; ^6^ aspartate transaminase.

**Table 2:** Assay values before and after vitamin K1 administration presented as median (IQR). *n* = 52.

Assay	Before	After	Reference Interval	Trend	*p*-Value
Standard Coagulation Assays					
APTT ^1^ (s)	31.5 (27–39.5)	31.5 (28.3–39)	26–33	−	0.81
Owren PT ^2^ (INR ^3^)	1.4 (1.3–1.6)	1.3 (1.2–1.4)	0.9–1.2	↓	<0.001
Quick PT (s)	13.7 (12.5–14.4)	12.1 (11.4–13.1)	10–13	↓	<0.001
Fibrinogen (g/L)	4.2 (3.2–4.7)	5 (4.3–5.9)	2–4	↑	<0.001
Vitamin K-dependent proteins					
dp-ucMGP ^4^ (pmol/L)	840 (600–1300)	580 (450– 660)	< 300	↓	<0.001
PIVKA-II ^5^ (AU/mL)	0.36 (0.27–0.56)	0.23 (0.11–0.37)	< 0.15	↓	0.016
Coagulation factor activity					
FII ^6^ (kIU/L)	0.61 (0.47–0.76)	0.70 (0.53–0.86)	0.7–1.5	↑	<0.001
FVII (kIU/L)	0.48 (0.34–0.57)	0.61 (0.51–0.78)	0.4–1.6	↑	<0.001
FIX (kIU/L)	0.94 (0.77–1.25)	1.14 (0.89–1.38)	0.8–1.5	↑	<0.001
FX (kIU/L)	0.62 (0.47–0.79)	0.72 (0.59–0.93)	0.7–1.54	↑	<0.001
Protein C (kIU/L)	0.65 (0.56–0.80)	0.68 (0.54–0.86)	0.7–1.3	−	0.11
Protein S (kIU/L)	0.56 (0.46–0.78)	0.62 (0.50–0.83)	0.65–1.4	↑	0.024
Thrombin generation assays					
TGA ^7^ RB ^8^ AUC (nM)	2400 (2200–2900)	2600 (2300–3000)	1500–2700	↑	0.006
TGA RC ^9^, AUC (nM)	2200 (2000–2600)	2400 (2200–2800)	1200–2600	↑	0.005
ROTEM ^10^					
CT ^11^ (s)	82 (76–97)	86 (75–93)	38–79	−	0.29
CFT ^12^ (s)	78 (59–99)	76 (64–94)	34–159	−	0.30
α angle (°)	74 (70–78)	75 (71–78)	63–83	−	0.19
MCF ^13^ (mm)	67 (60–72)	67 (62–74)	50–77	↑	0.008
ML ^14^ (%)	7 (3–10)	5 (2–10)	0–18	−	0.39
Other analyses and SOFA ^15^					
CRP ^16^ (mg/L)	99 (64–235)	167 (109–239)	<5	↑	0.01
Haemoglobin (g/L)	106 (98–113)	98 (93–103)	117–170	↓	0.002
Leukocytes (×10^9^/L)	11 (8.0–13)	10.5 (9.0–14)	3.5–8.8	−	0.73
Platelets (×10^9^/L)	173 (125–229)	164 (116–235)	145–387	−	0.23
SOFA	6 (4–9)	4.5 (3–7)	0	↓	0.005
Vitamin K1 in nutrition (µg) ^17^	32 (32–46)	NA	NA	NA	NA
Deficiency vitamin K1 in nutrition (µg) ^18^	88 (74–88)	NA	NA	NA	NA

^1^ Activated partial thromboplastin time; ^2^ Prothrombin time; ^3^ International normalized ratio; ^4^ Desphospho-uncarboxylated matrix Gla protein. Values are presented with 2 significant figures; ^5^ Protein induced by vitamin K absence/antagonist-II, median and quartiles are based on only six patients in which PIVKA-II was measurable; ^6^ Factor; ^7^ Thrombin generation assay. Values are presented with 2 significant figures.; ^8^ Reagent B; ^9^ Reagent C, for this analysis one sample was missing and calculation are based on 51 patients.; ^10^ Rotational thromboelastometry; ^11^ Clotting time; ^12^ Clot formation time; ^13^ Maximal clot firmness; ^14^ Maximal lysis; ^15^ Sequential organ failure assessment; ^16^ C-reactive protein; ^17^ Between sampling occasions—calculated intake through enteral or parenteral nutrition; ^18^ Between sampling occasions compared to the United States Food and Drug Administration recommended daily vitamin K1 intake of 120 µg per day; − Value unchanged between samplings; ↓ Value decreased to second sampling; ↑ Value increased to second sampling.

**Table 3:** Sensitivity analysis with a split dataset based on decreased or increased SOFA score.

Assay	Decreased SOFA ^1^ Score, *n* = 27			Increased or Stagnant SOFA Score, *n* = 25			*p*-Value Delta Changes ^3^
	Before	After	*p*-Value ^2^	Before	After	*p*-Value	
Standard coagulation assays							
APTT ^4^ (s)	35 (29–40)	32 (30– 42)	0.081	30 (28–40)	32 (28–41)	0.588	0.883
Qwren PT ^5^ (INR ^6^)	1.4 (1.3–1.6)	1.4 (1.2–1.5)	0.046	1.4 (1.4–1.6)	1.3 (1.2–1.4)	<0.001	0.027 ^7^
Quick PT (s)	14 (12– 15)	12 (12–13)	< 0.001	14 (12– 14)	12(11–13)	<0.001	0.963
Fibrinogen (g/L)	4.2 (3.2–4.5)	5.0 (4.1–5.5)	0.001	4.2 (3.3–4.9)	5.0 (4.4–6.4)	0.005	0.647
Vitamin K-dependent proteins							
dp-ucMGP ^8^ (pmol/L)	800 (600–1400)	560 (450–680)	0.001	970 (610–1270)	520 (410–660)	<0.001	0.589
PIVKA-II ^9^ (AU/mL)	-	-	-	-	-	-	-
Coagulation factor activity							
F ^10^ II (kIU/L)	0.61 (0.48–0.76)	0.68 (0.50–0.84)	0.011	0.62 (0.47–0.75)	0.73 (0.57–0.87)	0.002	0.203
FVII (kIU/L)	0.48 (0.39–0.56)	0.60 (0.50–0.79)	< 0.001	0.48 (0.31–0.68)	0.62 (0.51–0.81)	0.001	0.920
FIX (kIU/L)	0.89 (0.79–1.09)	1.01 (0.88–1.23)	0.001	1.06 (0.71–1.41)	1.35 (1.00–1.47)	0.001	0.436
FX (kIU/L)	0.53 (0.44–0.70)	0.66 (0.54–0.83)	0.001	0.66 (0.51–0.88)	0.88 (0.63–1.10)	<0.001	0.047 ^7^
Protein C (kIU/L)	0.60 (0.54–0.75)	0.64 (0.53–0.76)	0.775	0.70 (0.59–0.89)	0.79 (0.61–0.98)	0.013	0.041 ^7^
Protein S (kIU/L)	0.51 (0.44–0.59)	0.53 (0.47–0.63)	0.093	0.68 (0.56–0.85)	0.65 (0.56–1.00)	0.122	0.521
Thrombin generation assays							
TGA RB ^11^, AUC (nM)	2500 (2200–3000)	2600 (2500–3000)	0.121	2400 (2100–2900)	2600 (2300–3000)	0.020	0.405
TGA RC ^12^, AUC (nM)	2200 (2000–2500)	2400 (2300–2700)	0.052	2200 (2000–2600)	2300 (2100–2800)	0.049	0.706
ROTEM ^13^							
CT ^14^ (s)	82 (75–100)	87 (74–102)	0.801	82 (78–96)	84 (75–89)	0.045	0.181
CFT ^15^ (s)	79 (64–99)	76 (66–97)	0.461	76 (57–104	71 (59–92)	0.510	0.769
α angle (°)	74 (70–77)	74 (70–77)	0.282	74 (69–79)	75 (72–78)	0.531	0.854
MCF ^16^ (mm)	65 (60–71)	67 (62–73)	0.010	67 (57–74)	68 (62–74)	0.229	0.373
ML ^17^ (%)	7 (3–11)	6 (3–13)	0.220	5 (2–9)	5 (2–9)	0.760	0.373
Other analyses and SOFA							
CRP ^18^ (mg/L)	94 (64–234)	161 (119–218)	0.012	145 (56–275)	183 (102–262)	0.426	0.240
Haemoglobin (g/L)	108 (100–113)	96 (93–102)	0.001	105 (93–114)	101 (93–112)	0.162	0.047 ^19^
Leukocytes (×10^9^/L)	9.8 (8.5–12)	9.8 (8.5–12)	0.684	11 (8.6–15)	12 (9.6–14)	0.795	0.984
Platelets (×10^9^/L)	166 (122–231)	152 (120–289)	0.289	190 (125–228)	177 (115– 222)	0.455	0.984
SOFA	6 (4–10)	3 (2–5)	<0.001	4 (3–8)	6 (3–11)	0.005	<0.001

^1^ Sequential organ failure assessment; ^2^ Wilcoxon signed-rank test; ^3^ Mann–Whitney test; ^4^ Activated partial thromboplastin time; ^5^ Prothrombin time; ^6^ International normalized ratio; ^7^ Greater decrease in the group with increased SOFA score; ^8^ Desphospho-uncarboxylated matrix Gla protein. Values are presented with 2 significant figures; ^9^ Protein induced by vitamin K absence/antagonist-II; ^10^ Factor; ^11^ Thrombin generation assay, reagent B; ^12^ Thrombin generation assay, reagent C, for this analysis one sample was missing in the increased or stagnant SOFA score group, calculations are based on 24 patients for this analysis; ^13^ Rotational thromboelastometry; ^14^ Clotting time; ^15^ Clot formation time; ^16^ Maximal clot firmness; ^17^ Maximal lysis; ^18^ C-reactive protein; ^19^ Greater decrease in the group with decreased SOFA score.

## Data Availability

The datasets used and/or analyzed in the current study are available from the corresponding author on reasonable request.
